# Preparation of biologically activated lignite immobilized SRB particles and their AMD treatment characteristics

**DOI:** 10.1038/s41598-022-08029-y

**Published:** 2022-03-10

**Authors:** Junzhen Di, Yangyang Jiang, Mingjia Wang, Yanrong Dong

**Affiliations:** School of Civil Engineering, Liaoning University of Engineering and Technology, Fuxin, 123000 Liaoning Province China

**Keywords:** Microbiology, Environmental sciences, Chemistry

## Abstract

In response to the insufficient supply of carbon sources and the toxicity of heavy metal ions when using sulfate reducing bacteria (SRB) to treat acid mine wastewater (AMD), the immobilized particles are prepared with *Rhodopseudomonas*, SRB and lignite as the main raw materials. And based on single factor test and orthogonal test to determine the optimal ratio of biologically activated lignite fixed SRB particles. The adsorption characteristics of immobilized particles were studied under the optimal ratio, and the reaction kinetics and adsorption capacity of SRB particles immobilized on biologically activated lignite to different ions were analyzed. The results show that: lignite not only has good adsorption performance, but also can be used as the carbon source of SRB after being degraded by *Rhodopseudomonas*, solving the problems of low removal efficiency of SRB treatment of AMD and insufficient carbon source supply. When the dosage of lignite (particle size is 200 mesh), *Rhodopseudomonas*, and SRB are 3%, 10%, and 10% mesh, the prepared biologically activated lignite-immobilized SRB particles have the best effect on AMD treatment. The removal rates of SO_4_^2−^, Zn^2+^, and Cu^2+^ were 83.21%, 99.59%, and 99.93%, respectively, the pH was increased to 7.43, the COD release was 523 mg/L, and the ORP value was − 134 mV. The reduction process of SO_4_^2−^ by the biologically activated lignite-immobilized SRB particles conforms to the pseudo-first-order kinetics, and the adsorption of Zn^2+^ is more in line with the Freundlich isotherm adsorption equation and the pseudo-second-order kinetic model. And it does not spread in a single form, both internal and external diffusion occur. SEM, FT-IR, and BET analysis of biologically activated lignite immobilized SRB particles showed that the pore structure is developed, has a large number of adsorption sites, and some activated groups participate in the reaction. The adsorption process of Zn^2+^ and Cu^2+^ in AMD meets the multi-layer adsorption theory.

## Introduction

Mining activities make sulfide ore contact with oxygen to form AMD containing heavy metal ions (Zn^2+^, Cu^2+^, etc.) and high SO_4_^2−^ content^1^, which will have a serious impact on the environment^2,3^. At present, the most commonly used methods to deal with AMD mainly include physical, chemical and biological methods^4^. Shi Mingming and others used diatomaceous earth and bentonite as adsorbents to remove Zn^2+^, Pb^2+^ and Cd^2+^. The removal rates of diatomaceous earth for the three ions were 76.1%, 78.9% and 82.5%, respectively. The removal rates of these ions are 83.1%, 92.7% and 85.4%, respectively^5^. Although materials such as diatomaceous earth are used as adsorbents, the cost can be effectively reduced, but the effluent stability is poor^6^. After two-stage neutralization with sodium hydroxide, the removal rate of Fe, Mn, and Zn in the wastewater reached more than 99.7%^7^. The neutralization method is simple in process and low in cost, but the amount of sludge is large, the removal efficiency of high-concentration wastewater is low, and it is easy to cause secondary pollution to the environment^8^. After being treated by the Typha latifolia constructed wetland system, the average removal rates of COD, TSS, Pb, Zn, Cu and Cd were 92.19%, 99.62%, 93.98%, 97.02%, 96.87% and 96.39%, respectively^9^. However, the constructed wetland method covers a large area and is greatly affected by the seasonal climate^10^. The biological treatment of AMD has the advantages of low cost, strong applicability, and no secondary pollution. Therefore, it has been widely studied. SRB is the dominant strain in the biological method^11,12^. Using bagasse as the SRB carbon source to treat AMD, the average removal rates of SO_4_^2−^, Fe^3+^, Mn^2+^, Cr^6+^ and Cr^3+^ are 61.63%, 99.81%, 72.35%, 96.8% and 100%, respectively^13^. The SRB process removes various pollutants in AMD The rate is better than other processes. Metal ions such as Cu^2+^ and Zn^2+^ are soluble in the form of sulfate, but insoluble in the form of metal sulfide^14^. SRB is a type of microorganisms that use sulfate as a terminal electron acceptor for anaerobic respiration. They are involved in the sulfur and carbon cycles. Plays a vital role^15^. SRB oxidizes low-molecular-weight organic compounds and converts the sulfate in AMD into hydrogen sulfide. Hydrogen sulfide fixes the metal ions in AMD in the form of metal sulfide precipitation^16,17^. However, in the actual treatment process, due to the inhibition of AMD's low pH, the toxicity of heavy metal ions and the lack of carbon sources in the later stage, the effect of SRB on AMD treatment is not ideal^18^. According to reports, microbial immobilization technology can reduce the toxic effects of heavy metal ions and acidity on SRB, but it is still necessary to add the carbon source required for the growth of SRB when preparing immobilized particles^19^. Therefore, the need to find a new type of carbon source material as the main raw material for preparing SRB immobilized particles is a bottleneck in the prior art.

Lignite has abundant reserves in China, accounting for 13% of the country's coal resources. Most of them are located in the shallow layer of the surface, which is convenient for mining^20^. Lignite is rich in humic acid^21^. Humic acid in lignite is a negatively charged polyelectrolyte, which adsorbs heavy metal ions in AMD through electrostatic adsorption^16^. The developed pore structure and specific surface area of lignite provide abundant active sites for heavy metal ions^22^. Lignite is rich in oxygen-containing functional groups, such as carboxyl, alcoholic hydroxyl, phenolic hydroxyl, quinone, carbonyl, and methoxy^22^, and heavy metal ions are easily combined with oxygen-containing functional groups on the surface of lignite. The active groups on the surface of lignite adsorb heavy metal ions through ion exchange and coordination. The surface of lignite is negatively charged^23,24^ and has a good affinity for H^+^ and heavy metal ions^25^. Lignite not only contains a large amount of organic matter, but also can treat heavy metal ions in AMD, and is suitable for assisting SRB to treat AMD. But SRB cannot use the macromolecular organic matter released by lignite as nutrients for growth and metabolism^26^. Therefore, it is necessary to degrade the macromolecular organic matter in lignite into small molecular organic matter to provide SRB.

*Rhodopseudomonas* is a type of bacteria that can metabolize hydrophobic compounds such as polycyclic hydrocarbons, coal and petroleum^12,27^, and has been widely used to degrade coal, petroleum, pesticides, etc.^11^. Liu Qian used *Rhodopseudomonas* to degrade activated carbon^28^. Zhang Jingyao's research shows that *Rhodopseudomonas* can degrade lignite^29^. However, the existing research is mostly focused on the degradation of macromolecular organic matter by *Rhodopseudomonas*, while the research on the reuse of degraded small molecular organics is relatively rare. In particular, research on using *Rhodopseudomonas* to degrade lignite to provide carbon source for SRB is rare. Using *Rhodopseudomonas* to degrade lignite as a filler for immobilization technology can effectively solve the problem of insufficient carbon sources for the growth of SRB.

Therefore, based on the embedding and cross-linking method, SRB, lignite, and *Rhodopseudomonas* are used as materials to prepare immobilized particles. Explore the optimal ratio of lignite as a mineral carbon source in the treatment of AMD with bio-activated lignite-immobilized SRB particles. Combining adsorption kinetics, adsorption isotherms and particle diffusion models, the reaction kinetics process of biologically activated lignite-immobilized SRB particles to SO_4_^2−^, Zn^2+^ and particle diffusion forms are analyzed. Reveal the mechanism of particle processing AMD and provide a new method for repairing AMD.

## Experiment method

### Single factor test and orthogonal test

According to the preparation method of immobilized particles^30^, the polyvinyl alcohol and sodium alginate that fully absorb water and swell by heating at 90 °C form a liquid gel. After the gel is cooled to room temperature, *Rhodopseudomonas*, SRB, and lignite are added to the gel. The above mixture was added dropwise to a 2% CaCl_2_ saturated boric acid solution at 100 r/min and stirred for 4 h. Wash the above particles with 0.9% normal saline to form biologically activated lignite-fixed SRB particles for use.

In the single factor experiment, the mass fraction of *Rhodopseudomonas*, lignite, SRB, and lignite particle size during the preparation of biologically activated lignite fixed SRB particles were selected as single factor factors. Among them, the mass fraction of *Rhodopseudomonas* is set to 0%, 10%, 20%, 30%, 40%, 50%, and the mass fraction of lignite is set to 1%, 3%, 5%, 7%. The mass fraction of SRB dosing is set to 10%, 30%, 50%, and the lignite particle size is set to 80 mesh, 100 mesh, and 200 mesh. The biologically activated lignite-immobilized SRB particles prepared under different experimental conditions were added to 300 mL of AMD according to the solid-to-liquid ratio of 1:10 (g/mL). Among them, the initial concentrations of SO_4_^2−^, Cu^2+^, Zn^2+^, Ca^2+^, and Mg^2+^ in AMD were 816 mg/L, 10 mg/L, 20 mg/L, 100 mg/L, 50 mg/L, and the initial pH value was 4.0. The conical flask containing biologically activated lignite-fixed SRB particles and AMD was placed in a SHZ-82 gas bath constant temperature oscillator at 30 °C and 150 r/min for a certain period of time, and then measure and analyze the SO_4_^2−^, Cu^2+^, Zn^2+^ removal rate and pH value, ORP (oxidation reduction potential) value, and COD concentration changes in wastewater.

On the basis of single factor test results, L_9_(3^4^) orthogonal test was performed on the above four factors to optimize the preparation conditions of biologically activated lignite immobilized SRB particles.

The concentration of SO_4_^2−^ is measured by V-1600PC visible spectrophotometer (HJ/T 342–2007), the concentration of Cu^2+^ and Zn^2+^ is measured by Z-2000 flame atomic spectrophotometer (GB/T 7475–2015), and the pH of the solution is measured by PHS-3C type pH meter (GB/T 6920–2015), COD content was measured with ET99730 COD meter (HJ/T399-2007), ORP value was measured with calomel electrode (SL 94-1994).

### Characteristic test

SO_4_^2−^ adsorption kinetics test: The biologically activated lignite-fixed SRB particles were added to 300 mL AMD (SO_4_^2−^ concentration is 816 mg/L, pH = 4) at a solid–liquid ratio of 1:10 (g/mL). Measure pH, COD, ORP and SO_4_^2−^ concentration after shaking 6 h, 12 h, 24 h, 48 h, 72 h, 96 h, and 120 h in SHZ-82 gas bath constant temperature oscillator at 30 °C and 150 r/min.

Zn^2+^ isotherm adsorption test: Weigh 3 g, 5 g, 10 g, 15 g, 20 g, 25 g biologically activated lignite fixed SRB particles and add them to 200 mL AMD (Zn^2+^ concentration 20 mg/L, pH = 4). Measure the Zn^2+^ concentration after shaking 3 h, 6 h, 9 h, 12 h, 24 h, 48 h, 72 h, 96 h and 120 h in SHZ-82 gas bath constant temperature oscillator at 30 °C and 150 r/min.

Zn^2+^ adsorption kinetics test: The biologically activated lignite fixed SRB particles were added to 300 mL AMD (Zn^2+^ concentration 20 mg/L, pH = 4) according to a solid–liquid ratio of 1:10 (g/mL), at 30 °C, 150 r/min SHZ-82 gas bath constant temperature shaker, respectively oscillate for 3 h, 6 h, 9 h, 12 h, 24 h, 48 h, 72 h, 96 h and 120 h and then measure the pH, COD, ORP and Zn^2+^ concentration.

## Test results and discussion

### Single factor test results and analysis

**Figure 1:**
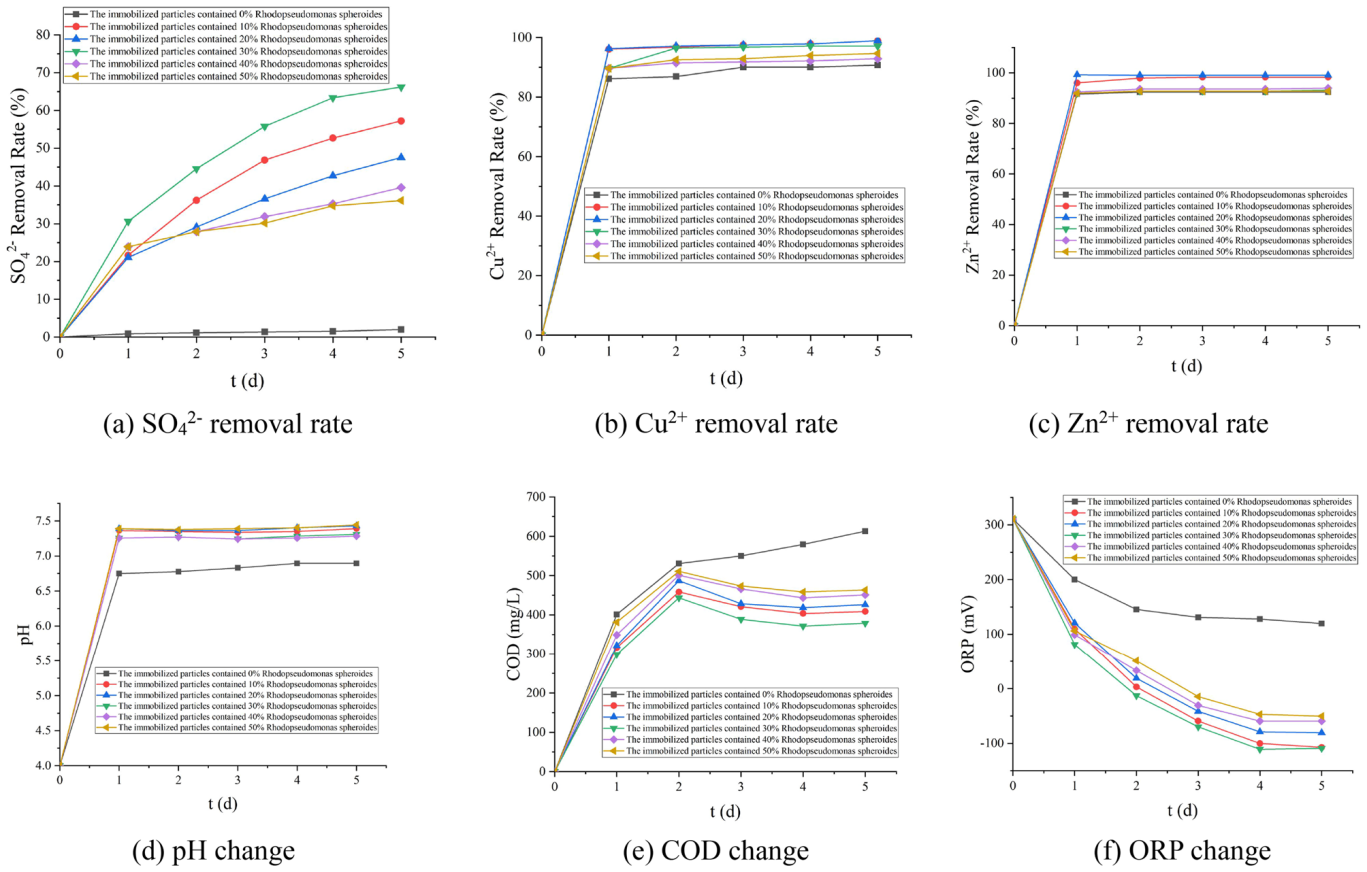
Effects of different dosages of Rhodopseudomonas on various indexes (200 mesh lignite with 5% mass fraction, 30% SRB concentrated bacteria liquid).

It can be seen from Fig. 1 that the biologically activated lignite-immobilized SRB particles prepared when the dosage of *Rhodopseudomonas* is 20% have the best removal effect on Cu^2+^ and Zn^2+^; When the dosage of *Rhodopseudomonas* is 30%, the removal effect of SO_4_^2−^ is the best. When the dosage of *Rhodopseudomonas* is in the range of 0–30%, the larger the dosage of *Rhodopseudomonas*, the better the treatment effect. *Rhodopseudomonas* degrades macromolecular organic matter in lignite into small molecular organic matter to provide carbon source for SRB and promote the SO_4_^2−^ reduction process^26,28^. When the dosage of *Rhodopseudomonas* is greater than 30%, the treatment effect on AMD becomes worse. When the lignite concentration is the same, the greater the concentration of *Rhodopseudomonas*, the worse the activity of *Rhodopseudomonas*^31^. Based on the above analysis, it can be concluded that the overall treatment effect is the best when the dosage of *Rhodopseudomonas* in the immobilized particles is 30%. Under this dosage, the removal rates of SO_4_^2−^, Zn^2+^, and Cu^2+^ were 66.3%, 97.1%, and 93.22%, respectively, the effluent pH was 7.32, the ORP in the solution was − 111 mV, and the release of COD was 379 mg/L.

**Figure 2:**
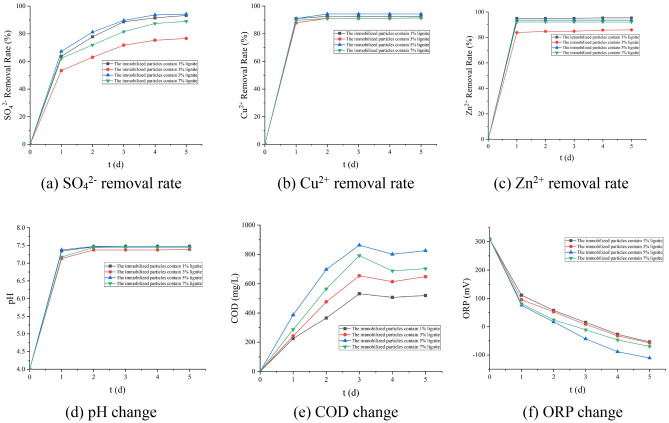
Effect of different dosage of lignite on various indexes (200 mesh brown coal, 30% Rhodopseudomonas, 30% SRB concentrated bacterial solution).

It can be seen from Fig. 2 that when the lignite dosage is 5%, the removal effect of SO_4_^2−^ and Cu^2+^ is the best, and the effect of increasing the pH of the solution is also the best. At this time, the removal rates of SO_4_^2−^, Zn^2+^, and Cu^2+^ are 94.44%, 94.6%, 93.65%, respectively. The effluent pH is 7.48, the ORP in the solution is − 110 mV, and the release of COD is 826 mg/L. The surface of lignite itself is negatively charged, and it is easy to adsorb positively charged metal ions^25^. The degradation of lignite can also provide a carbon source for the growth and metabolism of SRB^26,28^. Therefore, when the lignite dosage is between 0 and 5%, as the lignite dosage increases, the removal effect will gradually increase. Because the concentration of *Rhodopseudomonas* is constant, the continued addition of lignite will cause the excess lignite to be unable to be degraded and cannot be used effectively, and has little effect on the removal of AMD. Based on the above analysis, it can be obtained that the overall treatment effect is the best when the lignite dosage in the immobilized particles is 5%.

**Figure 3:**
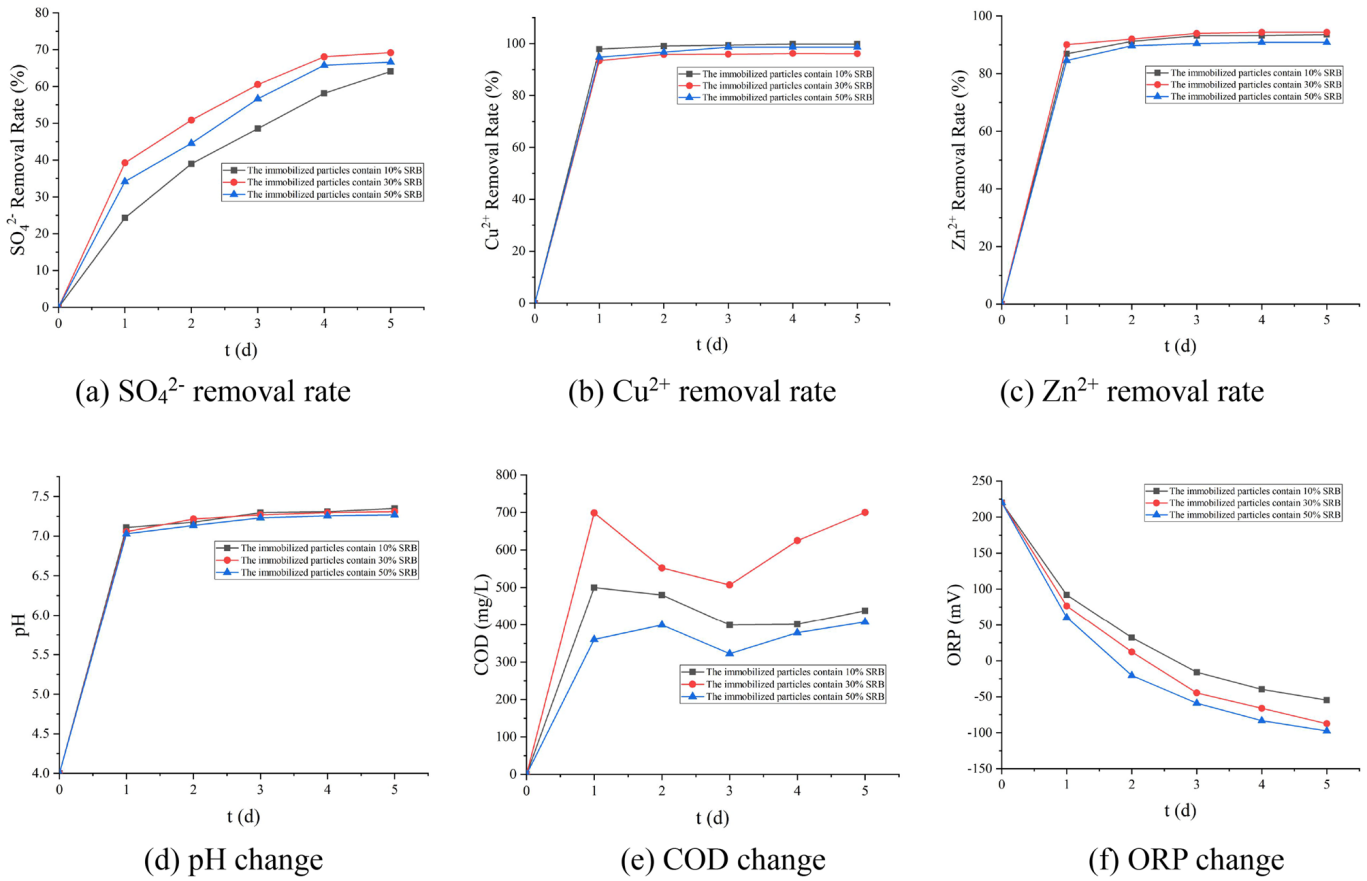
Effects of SRB dosage on various indexes (5% 200 mesh brown coal, 30% Rhodopseudomonas).

It can be seen from Fig. 3 that when the dosage of SRB is 30%, the removal effect of SO_4_^2−^ and Zn^2+^ is the best. Under this dosage, the removal rates of SO_4_^2−^, Zn^2+^, and Cu^2+^ are 68.92%, 96.1%, and 94.415%, respectively. The pH is 7.32, the ORP in the solution is − 97 mV, and the release of COD is 701 mg/L. The main mechanism that SRB can remove SO_4_^2−^ and Zn^2+^ is that SRB can effectively reduce SO_4_^2−^ to form sulfide, and combine with Zn^2+^ to form a precipitate, which removes Zn^2+^^17,18^.

**Figure 4:**
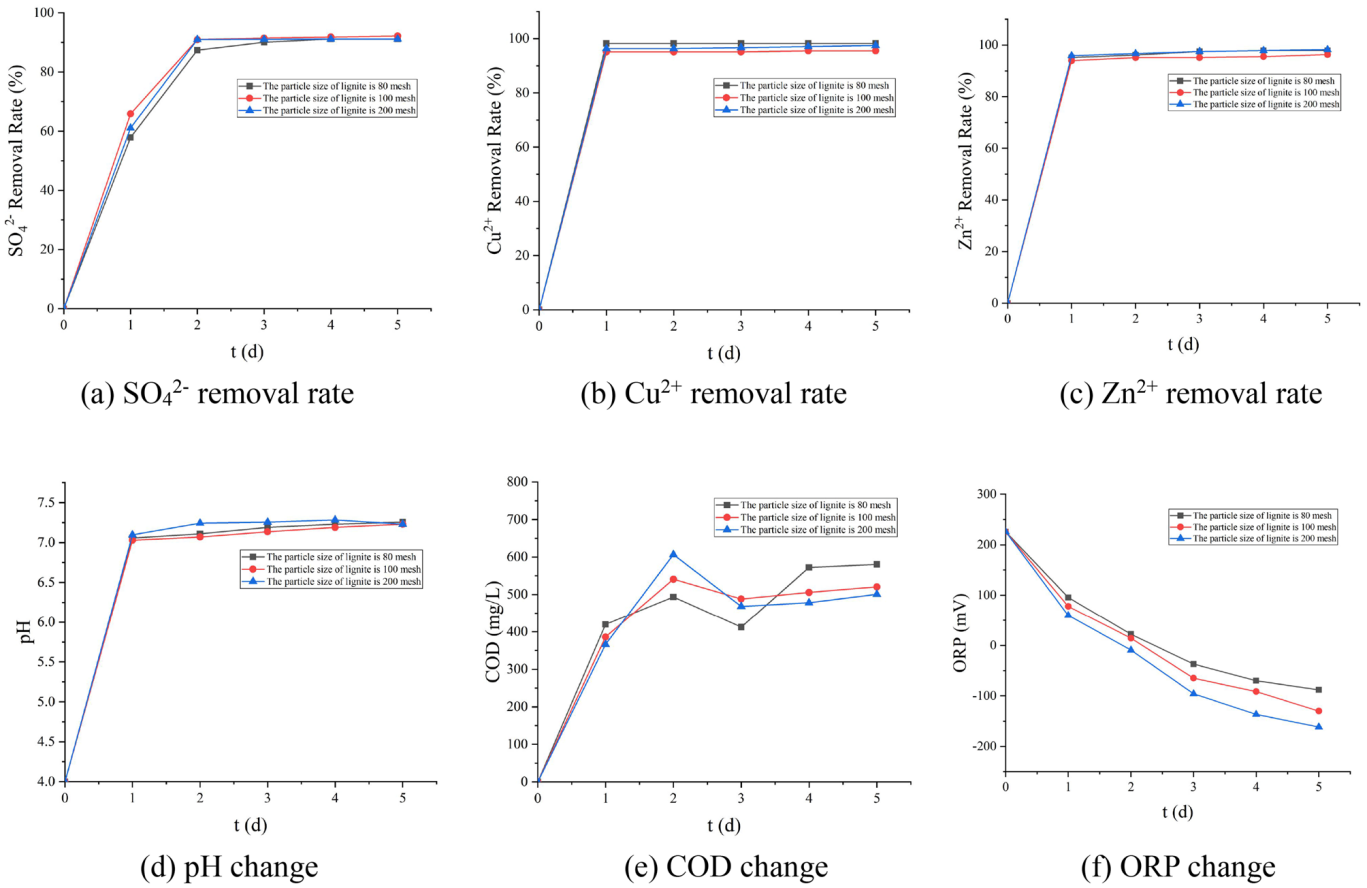
Effect of different particle size of lignite on various indexes (5% lignite, 30% Rhodopseudomonas, 30% SRB concentrated bacterial solution).

It can be seen from Fig. 4 that when the particle size of lignite is 80 mesh, the removal effect of Cu^2+^ is the best; when the particle size of lignite is 100 mesh, the effect of removing SO_4_^2−^ is the best; when the particle size of lignite is 200 mesh, the effect of removing Zn^2+^ and improving the pH of the solution is the best. Due to the developed pore structure, large specific surface area, and abundant oxygen-containing functional groups, lignite can effectively adsorb H^+^ and heavy metal ions in the system^22,23^. Therefore, the larger the particle size of lignite, the larger its specific surface area, and the better the removal effect of H^+^ and heavy metal ions. Based on the above analysis, it can be obtained that the overall treatment effect is the best when the lignite particle size of the immobilized particles is 200 mesh. Under this dosage, the removal rates of SO_4_^2−^, Zn^2+^, and Cu^2+^ were 91.04%, 97.64%, and 98.51%, respectively, the effluent pH was 7.32, the ORP in the solution was − 161 mV, and the COD release was 581 mg/L.

#### Orthogonal test results and analysis

The optimal dosage of each factor of the immobilized particles obtained from the previous single factor experiment (The dosage of *Rhodopseudomonas* is 30%; The dosage of lignite is 5%; The dosage of SRB is 30%; The diameter is 200 meshes), and the orthogonal test is carried out.

It can be seen from Table 1 that the order of influence of each factor on the SO_4_^2−^ removal effect is: A > D > C > B, and the analysis of variance is shown in Tables 2 and 3. The P values of these four factors and their effects are very low in this experiment (P < 0.05), indicating that the influence of each factor on the SO_4_^2−^ removal effect is not significant, which may be due to the relatively small degree of freedom of error in this experiment. The sensitivity of the orthogonal test results is reduced, which conceals the significance of the orthogonal test results.

**Table 1:** The L_9_(3^4^) orthogonal test and result.

Serial number	*Rhodopseudomonas* A/%	Lignite content B/%	SRB C/%	Particle size of lignite D/mesh	SO_4_^2−^ Test result	Cu^2+^ Test result	Zn^2+^ Test result	pH Test result	COD Test result	ORP Test result
1	10	3	10	80	80.96	99.18	96.77	7.13	499	− 134
2	10	5	30	100	77.70	99.93	95.58	7.28	482	− 129
3	10	7	50	200	82.28	99.18	99.59	7.42	516	− 123
4	30	3	30	200	75.17	98.81	98.28	7.31	511	− 119
5	30	5	50	80	71.77	96.57	95.66	7.23	479	− 124
6	30	7	10	100	72.21	95.59	96.58	7.23	485	− 120
7	50	3	50	100	81.49	93.05	94.67	7.43	516	− 119
8	50	5	10	200	83.21	95.59	97.12	7.35	521	− 114
9	50	7	30	80	75.21	94.74	95.00	7.36	523	− 116

**Table 2:** Intuitive analysis table.

Serial number	*Rhodopseudomonas* A	Lignite content B	SRB C	Particle size of lignite D
SO_4_^2−^ Mean value 1	80.311	79.207	78.793	75.980
SO_4_^2−^ Mean value 2	73.050	77.560	76.027	77.133
SO_4_^2−^ Mean value 3	79.970	76.567	78.513	80.220
SO_4_^2−^ Range	7.263	2.640	2.766	4.240
Cu^2+^ Mean value 1	99.430	97.013	96.787	96.830
Cu^2+^ Mean value 2	96.990	97.363	97.827	96.190
Cu^2+^ Mean value 3	94.460	96.503	96.267	97.860
Cu^2+^ Range	4.970	0.860	1.560	1.670
Zn^2+^ Mean value 1	97.313	96.573	96.823	95.810
Zn^2+^ Mean value 2	96.840	96.120	96.287	95.610
Zn^2+^ Mean value 3	95.597	97.057	96.640	98.330
Zn^2+^ Range	1.716	0.937	0.536	2.270
pH Mean value 1	7.277	7.290	7.237	7.240
pH Mean value 2	7.257	7.287	7.317	7.313
pH Mean value 3	7.380	7.337	7.360	7.360
pH Range	0.123	0.05	0.123	0.120
COD Mean value 1	499.000	508.667	501.667	500.333
COD Mean value 2	491.667	494.000	505.333	494.333
COD Mean value 3	520.000	508.000	503.667	516.000
COD Range	28.333	14.667	3.666	21.667
ORP Mean value 1	− 128.667	− 124.000	− 122.667	− 124.667
ORP Mean value 2	− 121.000	− 122.333	− 121.333	− 122.667
ORP Mean value 3	− 116.333	− 119.667	− 122.000	− 118.667
ORP Range	12.334	4.333	1.334	6.000

**Table 3:** Variance analysis.

Source of variance	*Rhodopseudomonas* /A	Lignite content /B	SRB /C	Particle size of lignite /D	Error /E	Sum
SO_4_^2−^ Sum of squares of deviations	100.760	10.668	13.916	28.835	10.670	164.849
SO_4_^2−^ Free Degree	2	2	2	2	2	8
SO_4_^2−^ Mean Square	50.38	5.334	6.958	14.4175	5.335	
SO_4_^2−^ F value	9.445	1.000	1.304	2.703		
SO_4_^2−^ P value	< 0.05	< 0.05	< 0.05	< 0.05		
SO_4_^2−^ Significant	⊙	⊙	⊙	⊙		
Cu^2+^ Sum of squares of deviations	37.055	1.122	3.786	4.259	1.12	47.342
Cu^2+^ Free Degree	2	2	2	2	2	8
Cu^2+^ Mean Square	18.5275	0.561	1.893	2.1295	0.56	
Cu^2+^ F value	33.026	1.000	3.374	3.796		
Cu^2+^ P value	> 0.05	< 0.05	< 0.05	< 0.05		
Cu^2+^ Significant	*	⊙	⊙	⊙		
Zn^2+^ Sum of squares of deviations	4.717	1.316	0.446	13.789	0.45	20.718
Zn^2+^ Free Degree	2	2	2	2	2	8
Zn^2+^ Mean Square	2.3585	0.658	0.223	6.8945	0.225	
Zn^2+^ F value	10.576	2.951	1.000	30.917		
Zn^2+^ P value	< 0.05	< 0.05	< 0.05	> 0.05		
Zn^2+^ Significant	⊙	⊙	⊙	*		
pH Sum of squares of deviations	0.026	0.005	0.023	0.022	0.01	0.086
pH Free Degree	2	2	2	2	2	8
pH Mean Square	0.013	0.0025	0.0115	0.011	0.005	
pH F value	5.200	1.000	4.600	4.400		
pH P value	< 0.05	< 0.05	< 0.05	< 0.05		
pH Significant	⊙	⊙	⊙	⊙		
COD Sum of squares of deviations	1297.556	411.556	20.222	750.889	20.22	2500.443
COD Free Degree	2	2	2	2	2	8
COD Mean Square	648.778	205.778	10.111	375.4445	10.11	
COD F value	64.166	20.352	1.000	37.132		
COD P value	> 0.05	> 0.05	< 0.05	> 0.05		
COD Significant	*	*	⊙	*		
ORP Sum of squares of deviations	232.667	28.667	2.667	56.000	2.67	322.671
ORP Free Degree	2	2	2	2	2	8
ORP Mean Square	116.3335	14.3335	1.3335	28	1.335	
ORP F value	87.239	10.749	1.000	20.997		
ORP P value	> 0.05	< 0.05	< 0.05	> 0.05		
ORP Significant	*	⊙	**⊙**	*		

F_0.05_ (2,2) = 19, F_0.01_ (2,2) = 99; *0. 05 ≤ P ≤ 0.01, significant; ^⊙^P < 0.05 was not significant.

It can be seen from Table 1 that the order of influence of various factors on the Cu^2+^ removal effect is: A > D > C > B, and the analysis of variance is shown in Tables 2 and 3. The results show that factor A has a significant effect on Cu^2+^ removal, while factors B, C and D have no significant effect on Cu^2+^ removal. The degradation of lignite by *Rhodopseudomonas* will increase the adsorption capacity of lignite for metal ions, so *Rhodopseudomonas* significantly affects the removal of Cu^2+^.

It can be seen from Table 1 that the order of influence of various factors on the Zn^2+^ removal effect is: D > A > B > C, and the analysis of variance is shown in Table 2 and Table 3. The results show that the removal effect of factors A, B and C on Zn^2+^ is not significant, and the removal effect of factor D on Zn^2+^ is significant. The surface of lignite is negatively charged and has a large specific surface area, so it exhibits excellent adsorption performance for positively charged metal ions. Therefore, the particle size of lignite has a significant effect on the removal of Zn^2+^.

According to Table 1, the order of influence of various factors on pH is: A = C > D > B, and the analysis of variance is shown in Tables 2 and 3. The results show that the influence of each factor on pH is not significant. This may be due to the relatively small degree of freedom of error in this experiment, which reduces the sensitivity of the orthogonal test results, which conceals the significance of the orthogonal test results.

It can be seen from Table 1 that the order of the influence of various factors on COD changes is: A > D > B > C, and the analysis of variance is shown in Tables 2 and 3. The results show that factors A, B and D have significant changes in COD, while factor C does not show significance. Lignite is an organic matter, which is a factor that can directly affect the change of COD content in the system. Therefore, the content and particle size of lignite in the system are factors that directly affect the change of COD content. *Rhodopseudomonas* converts macromolecular organics into small molecular organics by degrading brown coal. Therefore, *Rhodopseudomonas* also significantly affects COD changes in the system.

It can be seen from Table 1 that the order of the influence of each factor on the change of ORP is: A > D > B > C, and the analysis of variance is shown in Tables 2 and 3. The experimental results show that factors A and D show significant levels of ORP changes, and factors B and C show insignificant levels of ORP changes. *Rhodopseudomonas* will degrade the lignite in the system, and its activity can directly affect the ORP change of the system. The particle size of the lignite will determine the adhesion of the bacteria in the pores of the lignite, affect the activity of the bacteria in the system, and thus have an impact on the ORP.

According to the experimental results in Table 1, combined with intuitive analysis and variance analysis, the best combinations of SO_4_^2−^ removal rate, Cu^2+^ removal rate, Zn^2+^ removal rate, pH adjustment ability, COD release, and ORP value are A_1_B_1_C_1_D_3_, A_1_B_2_C_2_D_3_, A_1_B_3_C_1_D_3_, A_3_B_3_C_3_D_3_, A_3_B_1_C_2_D_3_, A_1_B_1_C_1_D_1_, respectively. After comprehensive consideration, it is finally determined that the best biologically activated lignite immobilized SRB particles is A_1_B_1_C_1_D_3_, that is, the dosage of *Rhodopseudomonas* is 10%, the dosage of lignite is 3%, and the dosage of SRB is 10% and the lignite particle size is 200 meshes. The biologically activated lignite-fixed SRB particles prepared under these conditions have the best effect on AMD treatment. After treatment, the SO_4_^2−^ removal rate is 83.21%, the Cu^2+^ removal rate is 99.93%, and the Zn^2+^ removal rate is 99.59%, pH increased to 7.43, COD release amount was 523 mg/L, ORP value was − 134 mV.

### Characteristic test results and analysis

#### The physical properties of biologically activated lignite immobilized SRB particles

**Figure 5:**
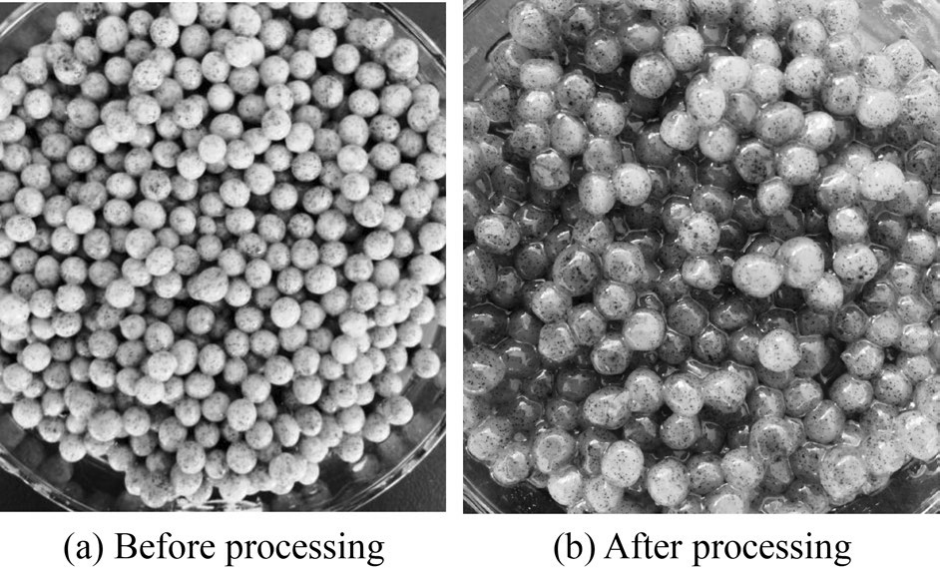
Granticle morphology of immobilized particles before and after AMD treatment.

It can be seen from Fig. 5a that the appearance of the particles before the treatment of AMD by the biologically activated lignite-fixed SRB particles is smooth and spherical, with hard texture, no obvious agglomeration, and a faint black inner core under the white coat. It can be seen from Fig. 5b that the bio-activated lignite-immobilized SRB particles after AMD treatment showed a full spherical shape, but the texture became softer and more hydrated, and the black core of the particles was clearly visible.

#### SO_4_^2−^ reduction kinetic analysis

The overall trend of the removal rate of SO_4_^2−^ by biologically activated lignite-fixed SRB particles increases gradually and then stabilizes. The reduction rate of SO_4_^2−^ is 12.283 mg/(L h) when the reaction is 1 day, the reduction rate is 0.840 mg/(L h) when the reaction is 5 days. SRB is a microorganism that uses sulfur oxides such as sulfate as an electron acceptor to obtain energy supply^32^. The test results show that the SRB in the biologically activated lignite-fixed SRB particles has a strong ability to adapt to the environment. The average reduction rate of SO_4_^2−^ within 1–4 days is 5.839 mg/(L h), which reflects that SRB can quickly enter a state of high growth rate and strong metabolism^33^. Within 1–4 days, *Rhodopseudomonas* degrades brown coal into organic matter that can be used by SRB^34,35^, which enhances the activity of microbial SRB and promotes the removal of SO_4_^2−^ by SRB. At the same time, lignite can also absorb heavy metal ions and H^+^ in the system, reduce environmental load, and reduce the stimulation of SRB caused by metal ions and over-acid conditions. Combined with the pH change curve of the solution, it can be seen that the system environment in the early stage of the reaction is too acid, which will inhibit the activity of SRB. When the pH value gradually rises to 7–8, the SRB activity is the best, but as the reaction continues, the nutrients and SO_4_^2−^ in the system are gradually consumed, so that the removal efficiency of SRB on SO_4_^2−^ reduced after 5 days.

**Figure 6:**
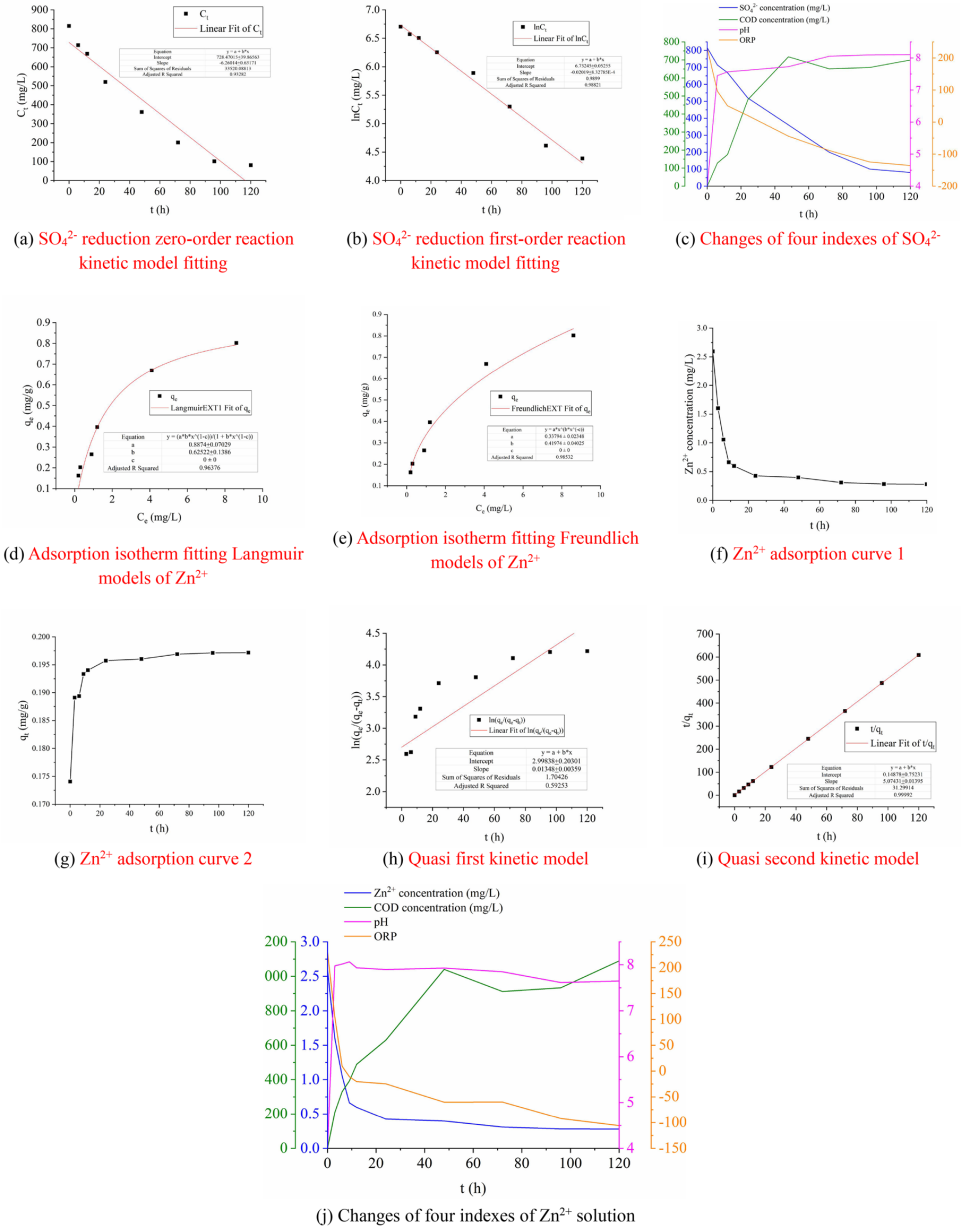
Fitting curves.

The zero-order kinetic model and the first-order kinetic model were used to analyze the adsorption kinetics process of biologically activated lignite immobilized SRB particles on SO_4_^2−^, and the fitting results are shown in Fig. 6a,b.

1 $$ C_{t} = C_{0} - k_{0} t $$

2 $$ \ln C_{t} = \ln C_{0} - k_{1} \cdot t $$

where, $C_{0}$ (mg/L) is the SO_4_^2−^ concentration at the initial moment; $C_{t}$ (mg/L) is the SO_4_^2−^ concentration at time *t*; $k_{0} (\text{mg}/\text{L}^{ - 1} \cdot h^{ - 1} )$ is Zero order reaction rate constant; $k_{1} (h^{ - 1} )$ is First order reaction rate constant.

From Fig. 6a,b, it can be seen that the *R*^2^ (0.98821) of the first-order reduction kinetic model is greater than the *R*^2^ (0.93282) of the zero-order reduction kinetic model, which indicates that the adsorption process of SO_4_^2−^ on the biologically activated lignite-fixed SRB particles is in line with first-order reduction kinetics better (The fitting equation is $y = { - 0}{\text{.02019}} \cdot x + {8}{\text{.32785}}$, *R*^2^ = 0.98821). The reduction process of SO_4_^2−^ by biologically activated lignite-immobilized SRB particles is mainly electron acceptor^36^. It can be seen from Fig. 6c that as the reaction progresses, the pH gradually increases from 4.0 and then stabilizes, and finally becomes weakly alkaline (pH = 8.1). At the beginning, the peracid reaction conditions are not conducive to the growth and metabolism of SRB, and the activity of SRB is low. At this time, the pH of the solution increases mainly because the lignite inside the immobilized particles contains rich ionizable groups (RCOOH ⇌ RCOO^−^ + H^+^). It has good two-way pH adjustment ability^36^, but the ability of lignite to adjust pH is limited. When the pH value rises to a certain level, the physiological activities of SRB are no longer inhibited, and SRB starts to actively carry out the SO_4_^2−^ reduction reaction. The H^+^ in the solution provides electrons for SRB to reduce SO_4_^2−^, and the pH value increases significantly. In the late stage of the reaction, although the system pH is suitable for SRB growth and metabolism, the SO_4_^2−^ concentration in the system is low, the electron acceptor content is insufficient, and the demand for H^+^ is also reduced, so the pH value of the system eventually stabilizes. The overall release of COD showed a trend of first increasing, then a small decrease, and finally a small increase. The final release of COD was 700 mg/L. The above changes in COD release are related to the leakage of organic matter released by lignite and SRB biological metabolites in immobilized particles^37^. In the early stage of the reaction, the lignite was degraded by *Rhodopseudomonas*, causing the lignite to be degraded into organic matter, and a large amount of organic matter was leaked out. However, the initial SRB was not enough to utilize such abundant organic matter, so the organic matter in the solution gradually accumulated and the COD content increased. With the progress of the reaction, the activity of SRB and the ability to utilize organic matter increase, and the organic matter in the solution is gradually consumed by SRB, so the organic matter content has a downward trend. In the late stage of the reaction, the activity of SRB and the ability to use organic matter decrease, resulting in a small accumulation of COD in the system. The overall ORP value gradually decreased and then stabilized, and the final ORP was − 136 mV. In the early stage of the reaction, lignite can be used as an electron donor for SRB. The lignite degraded by *Rhodopseudomonas* provides nutrients for the growth and metabolism of SRB, which further enhances the activity of SRB and reduces the ORP value. In the late stage of the reaction, due to the limitation of carbon source and electron acceptor content, the proliferation activity of SRB is inhibited and its activity is low, so the ORP value tends to be stable.

#### Analysis of the isotherm adsorption equation of Zn^2+^ on SRB particles immobilized by biologically activated lignite

According to formula (3), the adsorption capacity of Zn^2+^ by the biologically activated lignite-fixed SRB particles was calculated, and the Langmuir and Freundlich^38^ isotherm adsorption equations were used to fit the adsorption process of Zn^2+^ by the bio-activated lignite fixed SRB particles. The fitting results are shown in Fig. 6d,e.

3 $$ q_{e} = \frac{{\left( {C_{0} - C_{e} } \right) \cdot V}}{M} $$

where, $q_{e}$ (mg/g) is the equilibrium adsorption capacity of particles to Zn^2+^; $C_{0}$ and $C_{e}$ (mg/L) are the initial concentration and equilibrium concentration of Zn^2+^ in the solution, respectively; $V(L)$ is the Zn^2+^ solution volume; $M(g)$ is the dosage of particles.

4 $$ q_{e} = \frac{{b \cdot q_{\max } \cdot C_{e} }}{{1 + b \cdot C_{e} }} $$

5 $$ q_{e} = k \cdot C_{e}^{\frac{1}{n}} $$

where, $q_{e}$ (mg/g) is the equilibrium adsorption capacity of particles to Zn^2+^; $C_{e}$ (mg/L) is the equilibrium concentration of Zn^2+^ in the solution; $q_{\max }$ (mg/g) is the maximum theoretical adsorption capacity of particles on Zn^2+^; $b$ is a constant, related to the enthalpy of the adsorption reaction, expressed as the reciprocal of the concentration; $k$ is the Freundlich adsorption coefficient; $n$ is a constant, indicating the relative size of the unevenness of the adsorbent surface and the adsorption strength, usually greater than 1.

From Fig. 6d,e, it can be seen that the *R*^2^ (0.98532) of the Freundlich isotherm adsorption equation is greater than the *R*^2^ (0.96376) of the Langmuir isotherm adsorption equation, indicating that the adsorption process of the biologically activated lignite-immobilized SRB particles on Zn^2+^ in AMD is more consistent with the Freundlich isotherm Adsorption (The fitting equation is $q_{{\text{e}}} = {0}{\text{.32156}}C_{{\text{e}}}^{{{0}{\text{.41701}}}}$, *R*^2^ = 0.98821), the adsorption of Zn^2+^ by the biologically activated lignite-immobilized SRB particles is dominated by bilayer adsorption. 1/*n* is equal to 0.41974, greater than 0.1, less than 0.5, which is an index used to describe the heterogeneity of the adsorbent surface and the adsorption strength of the adsorbent in the Freundlich isotherm adsorption equation. This shows that the biologically activated lignite immobilized SRB particles have strong heterogeneity^39^ and the process of Zn^2+^ adsorption is easier to occur^40,41^. The Freundlich isotherm adsorption equation is fitted to obtain the saturated adsorption capacity of Zn^2+^ on the biologically activated lignite immobilized SRB particles is 0.789 mg/g. It is slightly different from the experimentally measured equilibrium adsorption capacity of Zn^2+^ (0.759 mg/g) of the biologically activated lignite-fixed SRB particles, indicating that the fitting result is more accurate.

#### The adsorption kinetics analysis of Zn^2+^ on the biologically activated lignite immobilized SRB particles

The Langergren pseudo-first-order kinetic Eq. (6) and McKay pseudo-second-order kinetic Eq. (7) were used to analyze the kinetic process of Zn^2+^ adsorption by biologically activated lignite-immobilized SRB particles. The fitting results are shown in Fig. 6f–i.

6 $$ \ln \left( {q_{e} - q_{t} } \right) = \ln q_{t} - k_{1} \cdot t $$

7 $$ \frac{t}{{q_{t} }} = \frac{1}{{k_{2} \cdot q_{e}^{2} }} + \frac{t}{{q_{e} }} $$

where, $$q_{e}$$ (mg/g) is the amount of adsorption at equilibrium; $$q_{t}$$ (mg/g) is the amount of adsorption at the time of adsorption time *t*; $$k_{1}$$ $$(\min^{ - 1} )$$ is the rate constant of quasi-first order kinetic reaction; $$k_{2} $$ (g/mg min) is the rate constant of pseudo-second-order kinetic reaction; $$h$$ (mg/(g min) is the initial adsorption rate, $$h = k_{2} \cdot q_{e}^{2}$$.

Intra-particle diffusion (IPD) and Elvoich kinetic models are used to determine the diffusion mechanism of Zn^2+^ on the biologically activated lignite-fixed SRB particles, see Eqs. (8) and (9).

8 $$ q_{t} = k_{i} t^{1/2} + C $$

9 $$ q_{t} = \alpha + \beta \ln t $$

where, $q_{t}$ (mg/g) is the amount of adsorption at the time of adsorption time *t*; $k_{i}$(mg min^1/2^/g) is the intraparticle diffusion rate constant; $C$ is the intercept reflecting the thickness range of the boundary layer; $\alpha$ and $\beta$ are the parameter model constants of the Elvoich equation.

It can be seen from Fig. 6h,i that the pseudo-second-order kinetic regression coefficient *R*^2^ (0.99992) of the adsorption of Zn^2+^ by the biologically activated lignite-fixed SRB particles is greater than the pseudo-first-order kinetic regression coefficient *R*^2^ (0.59253), and the regression coefficient *R*^2^ of pseudo-second-order kinetics is closer to 1. It shows that the pseudo-second-order kinetic equation $(y = {5}{\text{.07431}} \cdot x + {0}{\text{.01396)}}$ can more accurately describe the adsorption behavior of biologically activated lignite immobilized SRB particles on Zn^2+^^42^. It shows that the adsorption process of biologically activated lignite-immobilized SRB particles on Zn^2+^ is dominated by chemical adsorption, and the influence of chemical adsorption is higher than the influence of mass transfer step control^43–45^. It can be seen from Fig. 6j that when the biologically activated lignite immobilized SRB particles adsorbed Zn^2+^, the pH value of the solution as a whole showed a trend of increasing first and then stabilizing. In the initial stage of the reaction, the lignite with negatively charged surface easily attracts positively charged H^+^, which reduces the H^+^ content in the solution, so the pH value of the solution increases. As the pH of the solution gradually approached neutrality, the activity of SRB in the particles also increased. SRB reduction of SO_4_^2−^ also became a way to consume H^+^, and the pH of the solution gradually increased. However, as the reaction continued, the vacant sites on the surface of the lignite were gradually occupied by metal ions and H^+^, resulting in a decrease in its adsorption capacity. In addition, the H^+^ concentration in the solution is low, and the ability to provide electrons for SRB is also weak. Therefore, the H^+^ concentration in the system does not change substantially, and the pH of the solution tends to be stable. The overall change of COD presents a trend of first rising, then falling and then rising, and the final cumulative release of COD is 1090 mg/L. At the beginning of the reaction, *Rhodopseudomonas* degrades lignite, and COD gradually increases. As the reaction progresses, SRB consumes the degraded lignite as a carbon source for its own proliferation, and the COD content of the solution decreases. In the late stage of the reaction, the growth and metabolism capacity and activity of SRB are limited by the carbon source and terminal electron acceptor content, and COD accumulates again. The change of ORP value decreased first and then stabilized with the increase of reaction time. The final ORP value of the system was − 106 mV. The surface of lignite is negatively charged, and has obvious adsorption effect on positively charged heavy metal ions and H^+^, which reduces the toxic effect of acid and heavy metal ions in AMD on SRB, and the activity of SRB is enhanced. The brown coal degraded by *Rhodopseudomonas* provides nutrients for the growth and metabolism of SRB, and the activity of SRB is further enhanced. When the reaction progressed to the later stage, the lignite basically reached adsorption saturation, the H^+^ concentration in the solution did not change significantly, and it provided fewer electrons for the SRB reduction process, the SRB activity was low, and the ORP value basically did not change.

**Figure 7:**
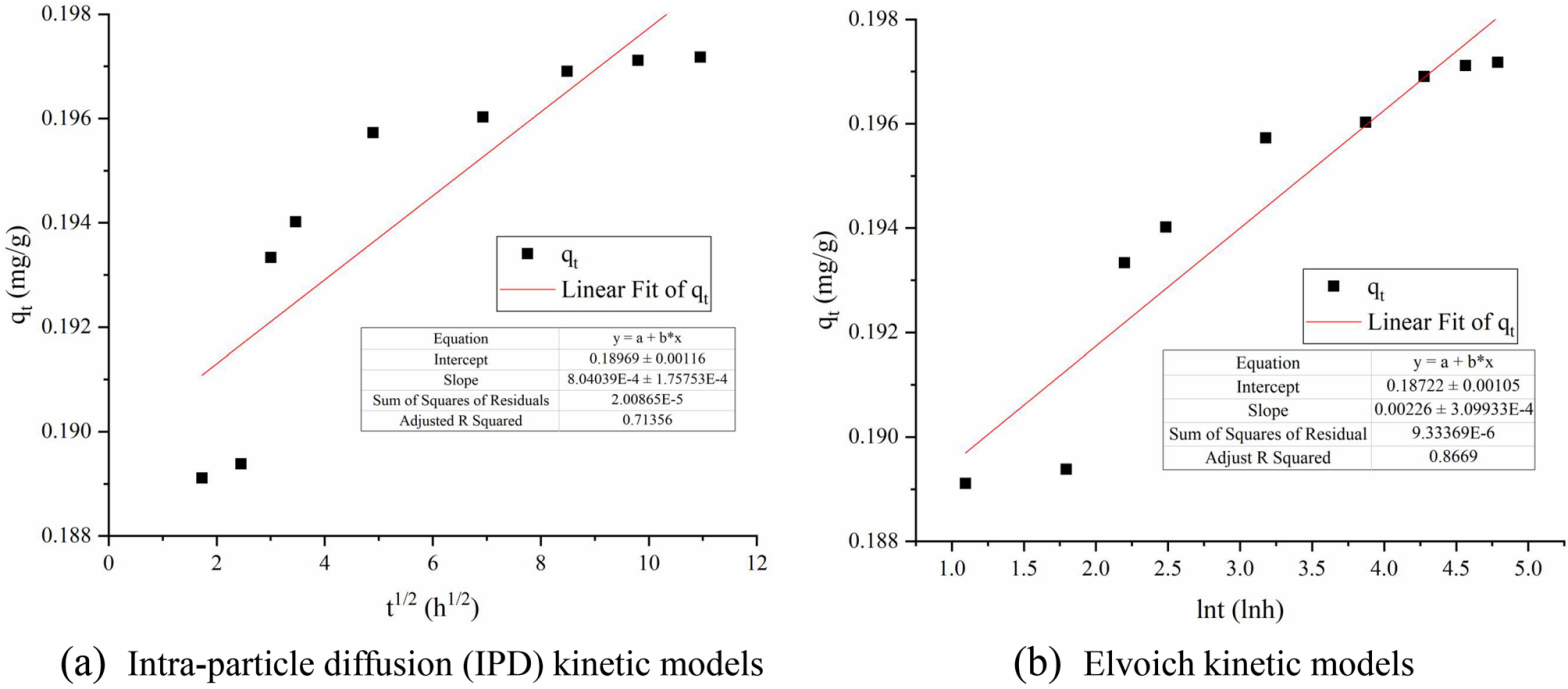
Intra-particle diffusion (IPD) and Elvoich kinetic models of biologically activated lignite immobilized SRB particles.

According to Fig. 7, before reaching the adsorption equilibrium, neither the internal diffusion nor the external diffusion curves cross the origin, indicating that the two diffusion types of particles are not the only rate control steps in the adsorption process^46,47^. The extra-particle diffusion model *R*^2^ = 0.8669 is greater than the intra-particle diffusion model, indicating that the extra-particle diffusion is dominant in the adsorption process.

## Characterization and analysis of AMD treatment with biologically activated lignite immobilized SRB particles

### Scanning electron microscope SEM analysis

**Figure 8:**
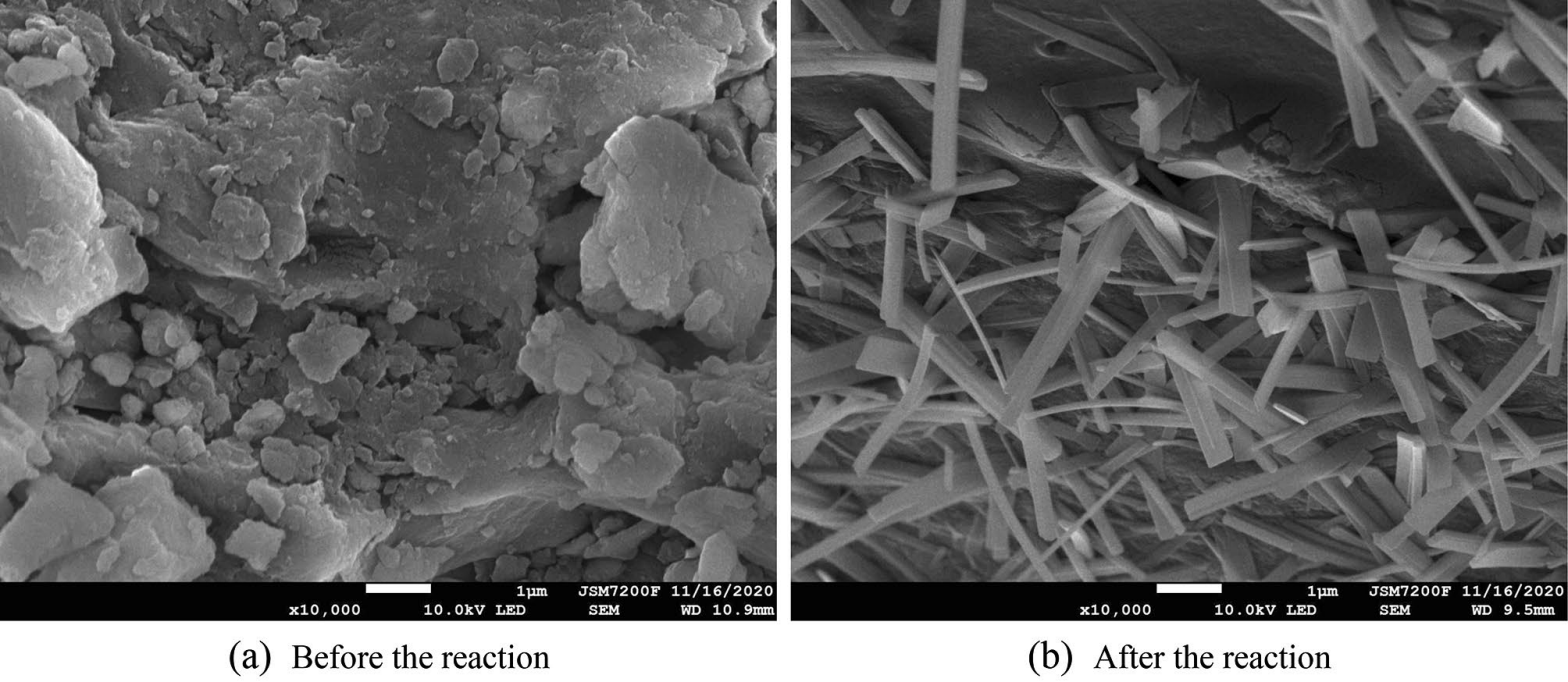
SEM analysis of bio-activated lignite fixed SRB particles before and after AMD treatment.

As shown in Fig. 8, the biologically activated lignite-immobilized SRB particles have a loose surface structure, uneven shape and size, and a developed pore structure before treating AMD, which can provide a large number of adsorption sites. After the AMD is treated, the surface structure is destroyed, the surface is wrinkled and cracked, and a large number of strips are formed. It shows that on the surface of lignite, Cu^2+^, Zn^2+^ react with SRB to metabolize SO_4_^2−^ the final product S^2−^ to produce metal sulfide precipitates. SRB uses the lignite degraded by *Rhodopseudomonas* as a carbon source for growth and metabolism.

### FT-IR infrared spectrum analysis

The lignite in the biologically activated lignite fixed SRB particles is the main component. The lignite is mainly composed of minerals such as quartz, and is rich in active groups such as –OH, –C=O, and –CHO. These active groups can undergo chemical reactions such as ion exchange and coordination complexation with metal ions.

**Figure 9:**
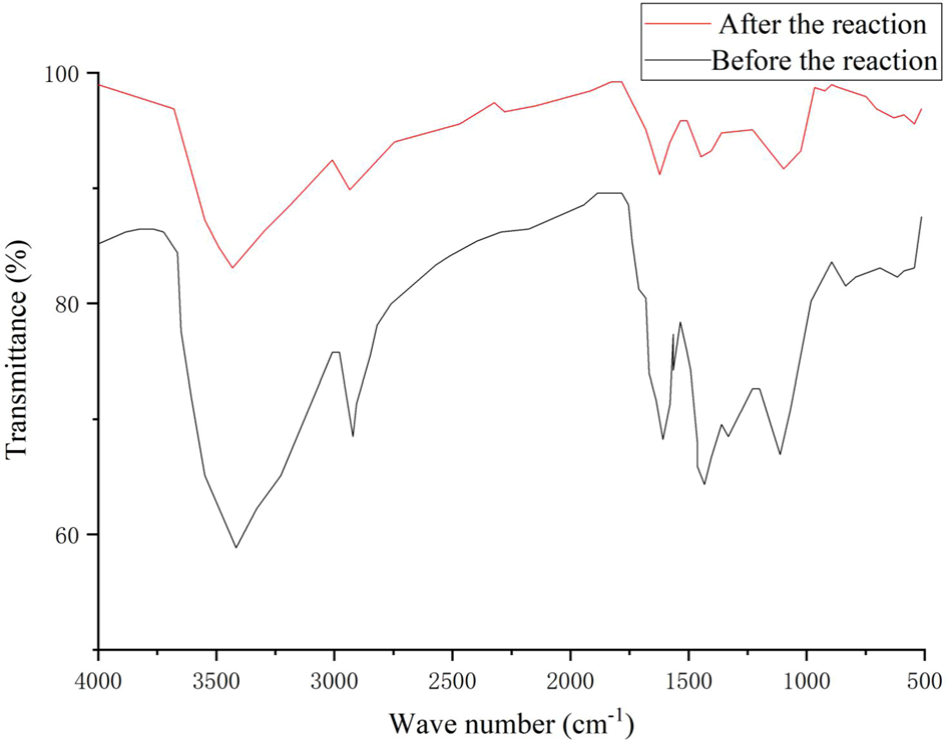
FT-IR spectra before and after the treatment of AMD with biologically activated lignite fixed SRB particles.

As shown in Fig. 9, the waveforms before and after the treatment of AMD with biologically activated lignite-fixed SRB particles were roughly the same, but the wave peaks were quite different. The increase of the corresponding OH stretching vibration absorption peak in alcohol and phenol at 3414 cm^−1^ indicates that the hydroxyl groups in alcohol and phenol coordinate with metal ions, and part of the hydroxyl groups are released in the form of water. At 2900 cm^−1^, the corresponding alkane C–H anti-symmetric stretching vibration combines with substituents at Cu^2+^ and Zn^2+^ to reduce the content of substituents. At 1615 cm^−1^, it corresponds to the C=C stretching vibration in the benzene ring. Under the degradation of *Rhodopseudomonas*, unsaturated macromolecular substances are degraded, so their content is reduced. In the range of 650–900 cm^−1^, the series of absorption peaks corresponding to the complex C–H out-of-plane bending vibration changes, and the structure of the biologically activated lignite-fixed SRB particles collapsed to a certain extent.

### BET analysis of bio-activated lignite immobilized SRB particles

It can be seen from Table 4 that the Langmuir specific surface area of the biologically activated lignite-immobilized SRB particles after the reaction with AMD is 1.53 times that before the reaction, and the BET specific surface area is 1.58 times that before the reaction. Because Langmuir theory believes that adsorbate molecules on the surface of the adsorbent is limited to single-layer adsorption, and BET theory believes that adsorbate molecules on the surface of the adsorbent are not limited to single-layer adsorption, but multi-layer adsorption^48^, so the two have big gap. And the Langmuir specific surface area before and after the reaction is obviously larger than the BET specific surface area. The BET correlation coefficient is greater than the Langmuir correlation coefficient, indicating that the adsorption process of biologically activated lignite immobilized SRB particles is more in line with the multilayer adsorption theory.

**Table 4:** The specific surface area before and after the treatment of AMD with biologically activated lignite immobilized SRB particles.

	Langmuir specific surface area (m^2^/g)	BET specific surface area (m^2^/g)	BET molecular cross-sectional area (nm^2^)	BET correlation coefficient	Langmuir correlation coefficient
Before the reaction	16.6545	7.4621	0.1620	0.9990	0.9860
After the reaction	25.5604	11.8146	0.1620	0.9990	0.9860

## Conclusion

In this study, the use of sulfate-reducing bacteria to treat acid mine wastewater is inhibited and toxic, and proposed to use *Rhodopseudomonas*, SRB, and lignite to make immobilized particles. Utilize single factor and orthogonal experiment experiment, adsorption kinetics, adsorption thermodynamics, particle diffusion model. Combine SEM, FT-IR, BET characterization. The mechanism of biologically activated lignite immobilized SRB particles to treat AMD was explored. Demonstrated the application potential of biologically activated lignite immobilized SRB particles in AMD treatment. Draw the following conclusions:

1. Single factor experiments show that the dosage of *Rhodopseudomonas* is 30%, the dosage of lignite is 5%, the dosage of SRB is 30%, and the particle size of lignite is 200 mesh, the biologically activated lignite-fixed SRB particles can treat AMD best.
2. On the basis of the single-factor experiment of the immobilized particle ratio in the early stage, based on the orthogonal experiment combined with the SO_4_^2−^, Cu^2+^, Zn^2+^ removal rate, pH improvement effect, COD release and ORP value and other index changes in the solution, the final determination of the best combination of bio-activated lignite fixed SRB particles is A_1_B_1_C_1_D_3_, that is, the dosage of *Rhodopseudomonas* in the immobilized particles is 10%, the dosage of lignite is 3%, the dosage of SRB is 10%, and the particle size of lignite is 200 mesh. Under this ratio, the removal rate of SO_4_^2−^ by the immobilized particles is 83.21%, the removal rate of Zn^2+^ is 99.59%, the removal rate of Cu^2+^ is 99.93%, the pH is increased to 7.43, the release of COD is 523 mg/L, and the ORP value is − 134 mV.
3. The reduction process of SO_4_^2−^ by the immobilized particles accords with the pseudo-first order kinetics, and the fitting equation is $y = - 0.02019 \cdot x + 6.73245$,$R^{2} = 0.98821$. The main factor affecting the reduction process is the electron acceptor.
4. The isotherm adsorption of Zn^2+^ by the immobilized particles is more in line with the Freundlich isotherm adsorption equation, and the fitting equation is $q_{e} = 0.32156 \cdot C_{e}^{0.41701}$,$R^{2} = 0.96532$. When the equilibrium concentration is 8.6175 mg/L, the equilibrium adsorption capacity can reach 0.759 mg/g. It shows that the lignite in the immobilized particles can adsorb Zn^2+^ easily, and the surface of the adsorbent is uneven; The adsorption kinetics of Zn^2+^ by lignite inside the immobilized particles is more in line with the pseudo-second-order kinetic model, and the fitting equation is $y = 5.07431 \cdot x + 0.14878$,$R^{{2}} = 0.99992$. It shows that the adsorption of lignite to Zn^2+^ is mainly chemical adsorption. The external diffusion model of immobilized particles *R*^2^ = 0.8669, which is greater than the internal diffusion model, but the two curves did not pass the origin, indicating that the particles do not diffuse in a single form, and the external diffusion is more dominant than the internal diffusion.
5. SEM, FT-IR, and BET analysis of the immobilized particles before and after the AMD treatment showed that the surface pores were developed. Cu^2+^, Zn^2+^ react with the substituents OH, C-H, etc., and react with SRB to metabolize SO_4_^2−^ the final product S^2−^ to form metal sulfide precipitates. This adsorption process belongs to multilayer adsorption.
